# Effect of Dental Follow-Up on Dental Consultation and Checkup Rates for Patients With Diabetes: A Multicenter Before–After Study

**DOI:** 10.7759/cureus.75145

**Published:** 2024-12-05

**Authors:** Shu Sekiya, Yasunari Mano, Tatsunori Suzuki, Rei Tanaka, Shumpei Harigai, Yoshiaki Shikamura

**Affiliations:** 1 Faculty of Pharmaceutical Sciences, Tokyo University of Science, Noda, JPN; 2 Community Pharmacy, Kaede Pharmacy, Noda, JPN; 3 Faculty of Pharmaceutical Sciences, Shonan University of Medical Sciences, Yokohama, JPN; 4 Community Pharmacy, Emuzu Pharmacy, Ashikaga, JPN

**Keywords:** community pharmacy, dental hygiene, diabetes mellitus, follow-up care, periodontal disease

## Abstract

Background

Patients with diabetes have an increased risk of developing periodontal diseases. Periodontal treatment also improves glycemic control. Therefore, regular dental consultations and checkups are important. Several collaborative efforts involving physicians, dentists, and pharmacists have been implemented to encourage dental consultations. Furthermore, pharmacists are legally required to provide follow-up after dispensing medications and to report medication adherence to physicians. However, reports on the involvement of community pharmacists in these initiatives remain limited; additionally, there are no reports focusing specifically on follow-up interventions related to oral health. Therefore, we aimed to create follow-up periodontal disease content for patients with diabetes patients and investigate the impact of follow-up by pharmacists on the dental consultation and checkup rates of patients with diabetes.

Methods

The study participants were patients with type 2 diabetes taking medication who visited pharmacies between November 1, 2022, and January 31, 2023, and provided their consent. We conducted a six-month follow-up using "FollowNavi^®^" (Unike Software Research Co., Ltd., Minato, Japan) and evaluated changes in dental consultation and check-up rates. A questionnaire survey was also conducted to investigate changes in awareness of dental hygiene and understanding of periodontal diseases. Furthermore, a questionnaire was issued to patients and pharmacists to investigate the user experience of "FollowNavi^®^".

Results

Follow-up was conducted in 113 patients. The dental consultation rate increased from 40.8% to 41.8%, and the dental checkup rate increased from 57.1% to 59.2%; however, the differences were not significant. The understanding of periodontal disease and awareness of prevention improved significantly after follow-up. In addition, the number of consultations on dental matters with pharmacists has increased significantly. However, there was no significant change in the HbA1c levels ​​before and after follow-up.

Conclusion

In a six-month follow-up using "FollowNavi^®^", there was no significant change in the dental consultation rate or examination rate. Nonetheless, oral care awareness and understanding improved according to the questionnaire. In addition, among patients who had not visited the dentist in the six months prior to participating in our study, a certain number of patients newly visited the dentist during the study period, suggesting that follow-up by community pharmacists may encourage dental visits. Furthermore, the number of consultations on dental matters with pharmacists increased. Our findings suggest the importance of follow-ups for pharmacists to become involved in community dental matters.

## Introduction

Interest in dental hygiene has recently increased in Japan. Accordingly, the Basic Policy on Economic and Fiscal Management and Reform 2024 promotes lifelong dental checkups (universal dental checkups).

Patients with diabetes are at a high risk of developing periodontal disease [1], which exacerbates poor glycemic control [2,3]. According to the 2011 Survey of Dental Diseases [4], > 60% of adults show some signs of periodontal disease and the number of patients with periodontal disease is estimated at approximately 70 million, with approximately only four million receiving ongoing treatment [5]. Therefore, improving the dental consultation rate among patients with diabetes who are at high risk of periodontal disease presents an urgent issue.

Several collaborative efforts involving physicians, dentists, and pharmacists have been implemented to encourage dental consultations; however, reports on the involvement of community pharmacists in these initiatives remain limited. Takaki et al. [6] examined the impact of a periodontal disease awareness campaign conducted at pharmacies on the dental consultation rate among patients with diabetes and reported that 8.2% of patients sought dental care after promotion. However, > 50% of the patients did not consult a dentist, highlighting the need for continuous guidance. Additionally, despite the short three-month duration of the study, approximately 20% of pharmacists reported it as a burden, and 35% stated that the three-month period was too long, raising concerns regarding the continuity of such efforts at pharmacies.

Under the Pharmaceutical and Medical Device Act, pharmacists are legally required to provide follow-up after dispensing medications and to report medication adherence to physicians. The effectiveness of follow-up in patients with diabetes has been demonstrated in several studies, including the present one [7-9]. However, there are no reports focusing specifically on follow-up interventions related to oral health.

Various approaches such as telephone and Information and Communication Technology (ICT) have been proposed to conduct follow-ups. LINE^®^, a widely used communication platform in Japan, has gained recognition as a follow-up tool [7,10,11], with the potential to reduce the burden on both patients and pharmacists.

Thus, we developed follow-up content on periodontal disease for patients with diabetes using the "FollowNavi^®^" platform (Unike Software Research Co., Ltd., Minato, Japan), a LINE^®^-based medication follow-up support tool. We aimed to investigate whether follow-up by community pharmacists could improve dental consultation rates, enhance the understanding of dental care, and raise awareness of oral hygiene among patients with diabetes.

## Materials and methods

Effectiveness survey

Study Design

This was a multicenter longitudinal before-after study. We evaluated the efficacy of dental follow-ups used by “FollowNavi^®^” for six months. In total, 113 individuals with type 2 diabetes mellitus (T2DM) were recruited from three pharmacies in Japan between November 2022 and January 2023.

Participants

Eligible participants were adults aged ≥20 years with T2DM who had a history of taking medication for more than two months at each pharmacy. Written informed consent was obtained from all the participants.

The first questionnaire was conducted before the participants participated in this study. Participants were excluded if they had type 1 diabetes mellitus, had no information on HbA1c within the last two months, or could not use a mobile phone. We also excluded participants who did not calculate the following guidance fees in the Japanese dispensing fee system: drug record administration, guidance fees from family pharmacists, and guidance fees related to home consultation services.

All pharmacists employed at the stores that were the subject of this study were eligible to participate, and informed consent was obtained when they completed the questionnaire.

Procedure

**Figure 1:**
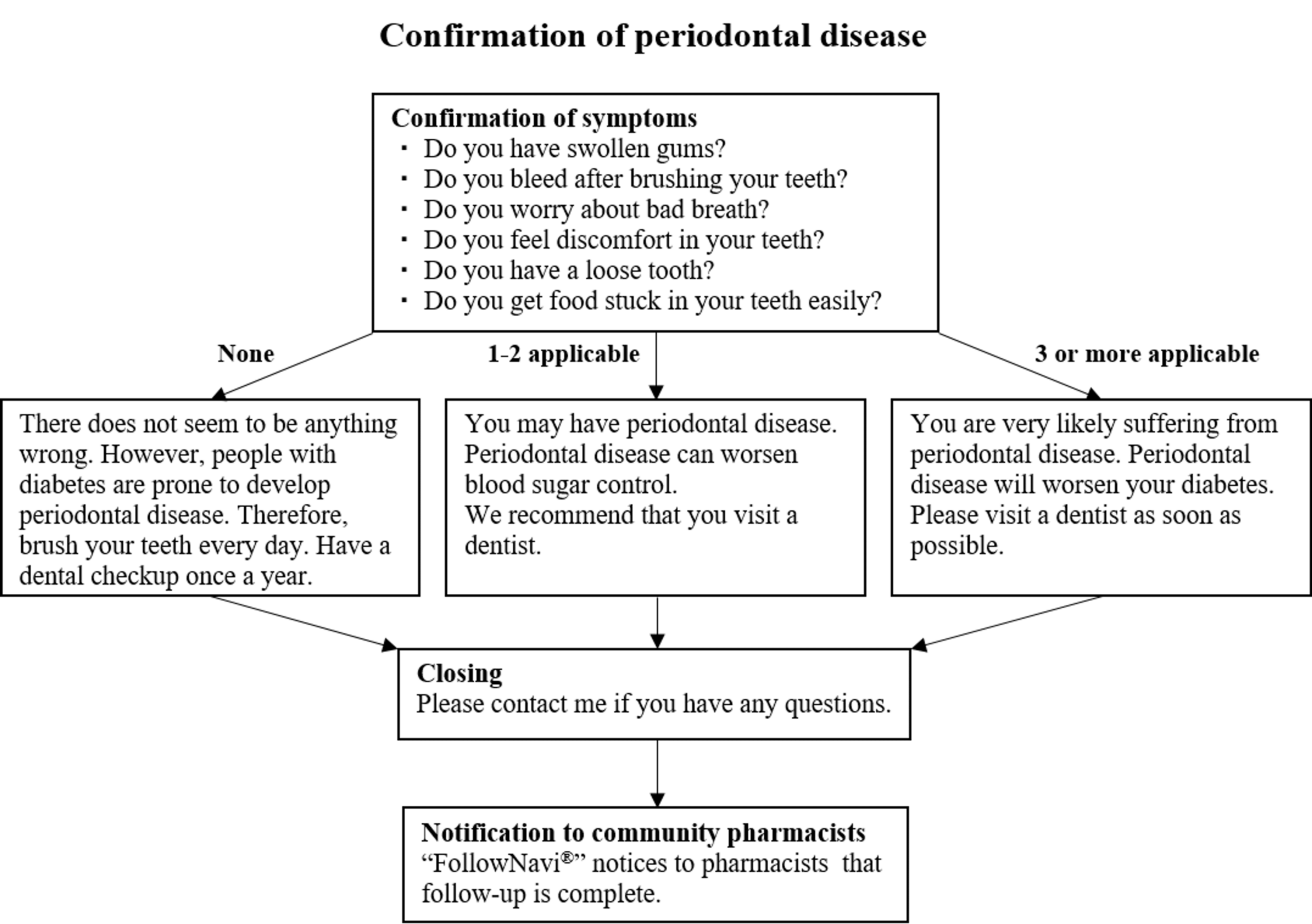
Example of the text messages about FollowNavi® for periodontal disease. The contents enable us to evaluate periodontal disease symptoms and whether the disease is present. FollowNavi^®^: Unike Software Research Co., Ltd., Minato, Japan

Patients who consented to follow-up were asked to register with the pharmacy’s LINE^®^ account, which was considered their registration for this study. A period of two weeks after registration, Content (A) - "Symptom check for suspected periodontal disease and encouragement to seek dental care" - was sent to participants, followed by Content (B) - "Educational information on diabetes and periodontal disease" - two months later. These contents were then alternated, with (A) and (B) sent every two months thereafter. To prevent follow-ups from becoming purely mechanical, free messages were also made available, allowing for personalized communication when necessary. Details of the follow-up are shown in Figure 1.

Outcome Measures

The primary outcomes were changes in dental consultation and checkup rates from baseline to six months. Data were collected at baseline and six months later from all participants. HbA1c levels of the patients were determined from the blood test results in the most recently recorded medical history during the six-month follow-up. Information regarding age, sex, and medication use was obtained from the medical history records. Furthermore, information regarding the duration of diabetes, dental consultations (for therapeutic purposes), participation in dental checkups (for cavity and periodontal disease screening, plaque removal, and brushing instruction), oral care status, willingness to prevent periodontal disease, and understanding of periodontal disease were evaluated using questionnaire surveys administered to patients.

Questionnaire survey

Questionnaires were administered to patients who received follow-ups and pharmacists who conducted the follow-ups. To assess the usability and acceptability of the system, both groups were asked regarding the clarity of the content, whether the number and frequency of questions were appropriate, the time required to respond, future expectations for the system, and any additional requests. Questionnaires were conducted at the end of the follow-up period. Consent was obtained from the respondents before they completed the questionnaire.

Statistical analysis

Data are presented as medians with interquartile ranges (IQR) or percentages. Missing data were imputed using the last-observation-carried-forward method. The Fisher’s exact test or McNemar’s test was used to compare categorical data. p < 0.05 was considered statistically significant. All statistical analyses were performed using IBM SPSS Statistics for Windows, Version 27 (Released 2020; IBM Corp., Armonk, New York, United States).

Ethical considerations

The study was approved by the ethics committee of the Tokyo University of Science (approval number: 20009).

## Results

Follow-up effectiveness

Patient Characteristics

This study included 113 patients (55 men and 56 women; 2 unspecified). The median age at baseline was 60 years (interquartile range [IQR]: 52-68), and the median HbA1c level was 6.7% (IQR: 6.3-7.0%). Overall, 26 patients (23.0%) had been diagnosed in less than 5 years, 85 patients (75.2%) had been diagnosed more than five years, and two patients (1.8%) did not provide an answer. The median follow-up period was 182 days (IQR: 125-266), and all patients underwent four follow-ups. The median number of responses to these follow-ups was 3.0 (IQR: 1.0-4.0). The median number of diabetes medications used was 3.0 (IQR: 2.0-4.0). The usage rates of diabetes medications were as follows: biguanides, 62.8%; sodium-glucose cotransporter 2 inhibitors, 51.3%; dipeptidyl peptidase IV inhibitors, 44.2%; glucagon-like peptide 1 receptor agonists, 31.0%; sulfonylureas, 16.8%; thiazolidinediones, 15.9%; insulin, 15.0%; α-glucosidase inhibitors, 13.3%; meglitinides, 12.4%; and imeglimin, 3.5% (multiple responses allowed) (Table 1).

**Table 1:** Demographic and baseline characteristics. Data are presented as median and interquartile range (IQR). † Two participants were excluded because they had no data. †† Five participants were excluded because they had no data. ††† Three participants were excluded because they had no data.

Demographic characteristics (n = 113)	Baseline
Age, years (IQR)^†^	60 (52–68)
Sex, no (%)	
Male	55 (48.7)
Female	56 (49.6)
No answer	2 (1.8)
HbA1c, % (IQR)	6.7 (6.3–7.0)
Follow-up period, day (IQR)^†^	182 (125–266)
Number of follow-ups, no (IQR)^††^	4.0 (4.0–4.0)
Number of replies to follow-up, no (IQR)^††^	3.0 (1.0-4.0)
Number of antidiabetic medications, no (IQR)^†††^	3.0 (2.0–4.0)
Use of hypoglycemic agent, no (%)	
Biguanides	71 (62.8)
Sodium-glucose cotransporter 2 inhibitors	58 (51.3)
Dipeptidyl peptidase-4 inhibitors	50 (44.2)
Imeglimin hydrochloride	4 (3.5)
Glucagon-like peptide-1 receptor agonists	35 (31.0)
Sulfonylurea agents	19 (16.8)
Thiazolidines	18 (15.9)
Insulin analogs	17 (15.0)
Alpha-glucosidase inhibitors	15 (13.3)
Rapid-acting insulin secretagogues	14 (12.4)
Duration of T2DM (years), no (%)	
<5	26 (23.0)
≧5	85 (75.2)
No answer	2 (1.8)

Changes in the Dental Consultation and Checkup Rates

The dental consultation and checkup rates at baseline and after six months are shown in Table 2. A total of 98 patients were included in the analysis, after excluding 15 patients for whom data at either baseline or six months were unavailable. The dental consultation rate increased from 40.8% to 41.8%, and the dental checkup rate increased from 57.1% to 59.2%; however, the changes were not statistically significant (P = 1.000, 0.824). HbA1c levels after six months were 6.8% (IQR: 6.4-7.2), with no significant change (P = 0.062).

**Table 2:** Changes in the dental consultation rate and dental checkup rate after dental follow-up by pharmacists. ^a^McNemar's test. Data are presented as median and interquartile range (IQR). † Fifteen participants were excluded because baseline data and/or six-month data were not available for them.

	Baseline	After six months	P-value
				Dental consultation rate (%)^†^	40.8	41.8	1.000^a^
Dental checkup rate(%)^†^	57.1	59.2	0.824^a^

Changes in Periodontal Disease Understanding

In the questionnaire, the question "Do you know that having periodontal disease can worsen blood sugar levels?," the number of patients who answered "Yes" increased from 64 (65.3%) at baseline to 85 (86.7%) (P < 0.001). In response to the question "Do you know that diabetes is a risk for periodontal disease?," 72 patients (73.5%) answered "Yes" at baseline, but 93 (94.9%) after six months (P < 0.001) (Table 3).

**Table 3:** Changes in understanding of periodontal disease and diabetes after dental follow-up by pharmacists. ^a^McNemar's test. Participants without available data at baseline and/or at six months were excluded from the analysis.

Variable	n	Baseline	After six months	P-value
					Understanding that periodontal disease worsens blood sugar levels, n (%)	98	64 (65.3)	85 (86.7)	<0.001^a^
Understanding that diabetes is a risk for periodontal disease, n (%)	98	72 (73.5)	93 (94.9)	<0.001^a^

Changes in Awareness of Oral Care

In response to the question, "Do you want to prevent periodontal disease (by consultation, the dentist, undergoing checkups, and brushing your teeth properly)?," the number of patients who answered "Strongly agree" or "Agree" increased significantly from 84 (86.6%) at baseline to 92 (94.8%) after six months (P = 0.039). In response to the question "Do you try to keep your teeth healthy?," 62 people (63.3%) answered "Yes" at baseline, and 64 people (65.3%) answered "Yes" after six months (P = 0.845) (Table 4).

**Table 4:** Changes in awareness about oral care after dental follow-up by pharmacists. ^a^McNemar's test. Participants without available data at baseline and/or at six months were excluded from the analysis.

Variable	n	Baseline	After six months	P-value
Willingness to prevent periodontal disease, n (%)	97	84 (86.6)	92 (94.8)	0.039^a^
Oral care practices, n (%)	98	62 (63.3)	64 (65.3)	0.845^a^

Changes in Pharmacist Consultations Regarding Dental-Related Matters

**Figure 2:**
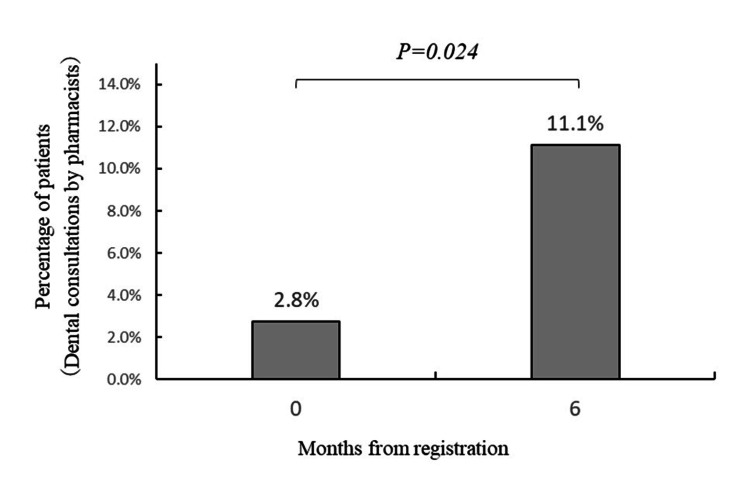
Changes in the proportion of patients who have consulted pharmacists regarding dental issues. Fisher's exact test was used.

In response to the question "Have you ever consulted a pharmacist about your teeth or mouth?", three people (2.8%) answered "Yes" at baseline, and 11 people (11.1%) answered "Yes" after six months (P = 0.024) (Figure 2).

Questionnaire survey

Results of the Patient Questionnaire Survey

The results of the patient survey conducted at the end of the follow-up period are presented in Table 5. In response to the question, "Did you consider visiting a dentist by message?," 46 patients (40.7%) answered "Strongly agree" and 39 patients (34.5%) answered "Agree." For the question, "Did you understand the text messages well?," 52 patients (46.0%) responded "Well understood," and 12 (10.6%) said "Understood.” Meanwhile, 2 (1.8%) responded "Slightly difficult, 6 (5.3%) said "Difficult," and 23 (20.4%) did not respond. When asked, "Did you solve your question with FollowNavi^®^," 71 patients (62.8%) stated they "No questions,” 11 (9.7%) answered that "Solved in another way" 6 (5.3%), and 3 (2.7%) were unable to resolve their questions, with 21 (18.6%) providing no answer.

**Table 5:** Acceptability and utility of follow-up with"FollowNavi®" in patients. FollowNavi^®^: Unike Software Research Co., Ltd., Minato, Japan

Did you consider contacting a dentist by message?	n (%)
Strongly agree	46 (40.7)
Agree	39 (34.5)
Disagree	10 (8.8)
Strongly disagree	3 (2.7)
No answer	15 (13.3)
Did you understand the text messages well?	
Well understood	52 (46.0)
Understood	12 (10.6)
Neutral	18 (15.9)
Slightly difficult	2 (1.8)
Difficult	6 (5.3)
No answer	23 (20.4)
Did you solve your question with "FollowNavi^®^"	
Solved with ‟FollowNavi^®^”	6 (5.3)
Solved in another way	11 (9.7)
No	3 (2.7)
No question	71 (62.8)
No answer	21 (18.6)
Was the number of questions appropriate?	
Small	5 (4.4)
Slightly small	1 (0.9)
Appropriate	80 (70.8)
Slightly large	4 (3.5)
Large	1 (0.9)
No answer	22 (19.5)
Was the frequency of questions appropriate?	
Low	3 (2.7)
Slightly low	1 (0.9)
Appropriate	81 (71.7)
Slightly high	4 (3.5)
High	1 (0.9)
No answer	23 (20.4)
How long did it take to answer the question?	
Within 5 min	67 (59.3)
5–10 min	20 (17.7)
10–20 min	2 (1.8)
20–30 min	0 (0.0)
Over 30 min	0 (0.0)
No answer	24 (21.2)
Do you want to continue using "FollowNavi^®^"	
Yes	64 (56.6)
No	25 (22.1)
No answer	24 (21.2)

Regarding the number and frequency of the questions, 80 (70.8%) and 81 (71.7%) patients, respectively, responded that they were "Appropriate." As for the time required to answer the questions, 67 (59.3%) indicated that it took "Within 5 min," and 20 (17.7%) answered "5-10 min." In response to the question, "Do you want to continue using FollowNavi^®^?," 64 patients (56.6%) answered "Yes," 25 (22.1%) said "No," and 24 (21.2%) did not respond.

Results of the Pharmacist Questionnaire Survey

The results of the pharmacist questionnaire surveys are presented in Table 6. Surveys were collected from eight pharmacists who implemented follow-ups using the FollowNavi^®^. When asked, "Have you been asked questions about dentistry before this trial?," three participants (37.5%) answered "Yes." In response to the question "Did you have any questions about dentistry during this trial?," two pharmacists (25.0%) answered "Yes."

**Table 6:** Results of the questionnaire survey for pharmacists regarding the usability and functionality of "FollowNavi®" FollowNavi^®^: Unike Software Research Co., Ltd., Minato, Japan

Did you use "FollowNavi®"	n (%)
Yes	8 (100)
No	0 (0)
Have you been asked questions about dentistry before this trial?	
Yes	3(37.5)
No	5 (62.5)
Did you have any questions about dentistry during this trial?	
Yes	2 (25.0)
No	6 (75.0)
Do you think the text messages are easy for patients to understand?	
Easy	0 (0)
Slightly easy	0 (0)
Neutral	0 (0)
Slightly difficult	6 (75.0)
Difficult	2 (25.0)
Did you get sufficient patient information?	
Yes	5 (62.5)
No	3 (37.5)
Was the number of questions appropriate?	
Small	0 (0)
Slightly small	0 (0)
Appropriate	7 (87.5)
Slightly large	1 (12.5)
Large	0 (0)
Was the frequency of questions appropriate?	
Low	0 (0)
Slightly low	1 (12.5)
Appropriate	7 (87.5)
Slightly high	0 (0)
High	0 (0)
Was ‟FollowNavi®” easy to operate?	
Easy	1 (12.5)
Slightly easy	3 (37.5)
Neutral	1 (12.5)
Slightly difficult	3 (37.5)
Difficult	0 (0)
How long did it take to check the responses of patients?	
Within 5 min	5 (62.5)
5–10 min	2 (25.0)
10–20 min	1 (12.5)
20–30 min	0 (0)
Over 30 min	0 (0)
Please select the expected effects of "FollowNavi®" (multiple answers allowed)	
Confirmation of patient symptoms	8 (100)
Improved adherence	5 (62.5)
Early detection of adverse drug reactions	5 (62.5)
Improving therapeutic effect	0 (0)
Reduction of medication instruction time	0 (0)
Others	0 (0)
Do you want to continue using "FollowNavi®"	
Yes	5 (62.5)
No	3 (37.5)

When asked, "Do you think the text messages are easy for patients to understand?," six pharmacists (75.0%) said "Slightly difficult" and two (25.0%) said, "Difficult.” When asked, "Did you get sufficient patient information?," five (62.5%) answered "Yes," and three (37.5%) said "No." The specific information they were unable to obtain included "patients' feelings, thoughts, and other non-text-based information."

Regarding the appropriateness of the number of inquiries and frequency of follow-ups, seven pharmacists (87.5%) responded that both were "appropriate." When asked about the usability of FollowNavi^®^, one (12.5%) responded "Easy,” three (37.5%) said "Slightly difficult," one (12.5%) answered "Neutral," and three (37.5%) said "Slightly easy."

When asked, "How long did it take to check the patient responses?," five pharmacists (62.5%) answered less than 5 min, two (25.0%) answered between 5-10 min, and one (12.5%) answered between 10-20 min. Regarding the expected effects of FollowNavi^®^ (multiple responses allowed), all eight pharmacists (100%) cited "Confirmation of patient symptoms," five (62.5%) cited "Improvement of adherence," and five (62.5%) mentioned "early detection of adverse drug reactions."

Finally, when asked, "Do you want to continue FollowNavi®," five pharmacists (62.5%) answered "Yes."

## Discussion

The dental consultation rate at baseline in this study was 40.8%, which is comparable to that in previous reports [12,13] indicating 47.8% and 44.0%. Similarly, the dental checkup rate was 58.6%, which was slightly higher than the previously reported rate of 46.8% [14], although not substantially different. Based on these comparisons, the population in this study is representative of a broad population of patients with diabetes.

Munenaga et al. [15] reported that in patients with T2DM moderate to severe periodontal disease, HbA1c levels improved significantly three months after periodontal treatment. Engebretson et al. [16] showed that in a meta-analysis of nine RCTs, periodontal treatment significantly reduced HbA1c levels by 0.36%. These studies were conducted on periodontal patients and on patients who had undergone dental treatment. In our study, the rate of dental consultation did not improve significantly, and not all patients had periodontal disease, so it seems that there was no improvement in HbA1c levels.

Regarding the questionnaire survey, the patients' understanding of the relationship between periodontal disease and glycemic control increased significantly, as well as their awareness that diabetes is a risk factor for periodontal disease. At baseline, 65.3% and 73.5% of the patients had this knowledge, and these rates increased significantly to 86.7% and 94.9%, respectively, after six months. This suggests that the follow-up of periodontal content by community pharmacists effectively enhances patient understanding.

In terms of motivation for oral care, the proportion of patients who answered "Strongly agree" or "Agree" to the question, "Do you want to prevent periodontal disease (by visiting the dentist, undergoing checkups, and brushing your teeth properly)?" significantly increased from 86.6% at registration to 94.8% after six months (P = 0.039). Additionally, in response to the question after follow-up "Did you consider visiting a dentist by message?", 46 patients (40.7%) answered "Strongly agree" and 39 (34.5%) answered "Agree." These results confirm that follow-ups improved patients' awareness and motivation for oral care.

Despite these changes in awareness, the actual consultation rate did not improve, possibly because behavioral changes take time. According to the transtheoretical model of health behavior change [17], there are five stages of behavior change: pre-contemplation (not yet acknowledging that there is a problem behavior that needs to be changed), contemplation (acknowledging that there is a problem but not yet ready, sure of wanting, or lacks confidence to make a change), preparation (getting ready to change), action (changing behavior), and maintenance (maintaining the behavior change). The survey results suggested that the willingness to visit dentists improved, indicating a transition from the pre-contemplation stage to the contemplation stage. However, for patients to move to actual behavioral changes, a shift from the contemplation stage to the preparation and action stages is necessary, and building a trusting relationship is considered crucial for this transition [18,19]. In the pharmacist's questionnaire, some respondents mentioned the challenge of "being unable to obtain non-verbal information, such as the patient's feelings and thoughts," highlighting the limitations of mechanical follow-ups. To build trust and promote behavioral change among patients, combining mechanical follow-ups with in-person consultations and guidance is necessary.

In addition, when asked "Have you ever consulted a pharmacist about your teeth or mouth?," the percentage of answering "Yes" significantly increased from 3.1% at baseline to 10.3% after six months. This suggests that follow-ups may lead to an increase in dental consultations at pharmacies. Given the current emphasis on the role of pharmacists in health support and dental-pharmaceutical collaboration [20], the indication that follow-up could promote pharmacists’ involvement in dental consultations is useful. However, the number of consultations remains insufficient. Thus, if further long-term follow-up is conducted, it may lead to a further increase in the number of consultations with pharmacists and an associated improvement in the rate of dental visits.

In the patient questionnaire survey regarding usability, 52 (46.0%) answered that the messages were "Easy," while 12 (10.6%) answered that they were "Slightly easy.” These values were lower than those obtained in our study using diabetes follow-up data [7]. This may be due to the high number of non-answers to this question (20.4 %). Given that a combined total of 7.1% of patients answered that it was "Slightly difficult" or "Difficult," the contents were adequately understood. Regarding the number of questions, 70.8% answered that it was "Appropriate,” and regarding the frequency of questions, 71.7% answered that it was "Appropriate,” indicating that the number was suitable. In this study, follow-ups were conducted only once every two months. In our previous report [7], follow-ups were planned for each consultation and conducted approximately once a month, with > 90% responding that this was appropriate. Although it is difficult to generalize the results because the participants and contents of follow-ups varied, the frequency of follow-up may not be an issue, depending on the patient. Therefore, pharmacists should consider medication and patient information and conduct frequent follow-ups.

Pharmacists often perform follow-ups on prescribed medications mainly to assess medication adherence, effectiveness, and side effects [21, 22]. Few studies report cases of pharmacies participating in dental care, which is not limited to follow-up care. Therefore, we believe that the results of this study, which confirmed the usefulness of dental follow-ups for patients with diabetes regardless of the prescribed medication, are significant when considering the scope of pharmacists' follow-up duties.

This study has two limitations. First, this study had a high rate of incomplete answers in the questionnaires. In the usability questionnaire, approximately 20% of the questions remained unanswered. Thus, improving the questionnaire method and questions is necessary for future surveys. Second, data, such as duration of diabetes, dental consultations, and participation in dental checkups, were obtained via questionnaire surveys administered to patients. Therefore, there is a possibility of recall bias and lack of accuracy. Further studies are needed to obtain accurate medical data and evaluate them.

## Conclusions

In the six-month follow-up using the periodontal contents of the "FollowNavi^®^" platform, which leverages LINE^®^ for the follow-up system, there was no significant improvement in the dental consultation, checkup rates, or HbA1C levels in patients with diabetes. However, the awareness and understanding of oral care improved, and there was an increase in dental consultations at pharmacies. The results of this study, which confirmed the usefulness of dental follow-ups for patients with diabetes regardless of the prescribed medication, are useful when considering the expansion of follow-ups in the future and ways of involvement in the dental field.

The findings show that pharmacists may be able to play a role in improving dental treatment for patients with diabetes through follow-up. This will be of great help in considering how community pharmacists can expand follow-up and become involved in the dental field.

## Appendices

Questionnaire

Patient Questionnaire Survey Before the Study (Japanese Version)

「フォロナビ」初回アンケート

「LINEによる歯周病予防等に関する情報提供および歯周病のチェック」を開始するにあたり、現在の状況について、アンケート調査をしたいと思います。つきましては、下記のアンケートにご回答をお願いします。

※本調査の結果は、学会等で発表する予定ですが、個人が特定できない形で公表します。

※このアンケートにご回答いただかなくても不利益はありません。

※同意は当薬局に申し出ることにより、いつでも撤回することができます。

※この調査内容をご理解され、ご協力に同意いただけますか？

（調査にご協力いただける方は、「同意します」の方に必ず☑を入れてください）

□同意しません・・・この調査にはご協力いただかなくて結構です。

□同意します　・・・以下の設問にご回答ください。　

以下の質問に対して、当てはまりものに☑（チェック）を付けてください。

1.     糖尿病の治療歴について、当てはまる年数をお選びください。

・ □1年未満　　□1年～5年未満　　□5年～10年未満　　□10年以上       

2.     現在、歯科受診をしていますか。

・□している（通院理由：　　　　　　　）　　□していない

3.     過去半年間に歯医者さんで検診（虫歯や歯周病の検査、歯垢除去、ブラッシングの指導）を受けたことがありますか。

・□受けた（頻度：　　回）　　　□受けていない

4.     今までに薬剤師に、歯や口の中に関する相談をしたことがありますか。

・□ある（内容：　　　　　　　　　　　　　　　　　　）　　　□ない

5.     歯を健康に保つことを心掛けていますか。

・□ある（具体的にやっていること：　　　　　　　　　　）　　□ない　

6.     歯周病があると血糖のコントロールが悪くなることを知っていますか。

・□知っている　　　□知らない

7.     糖尿病があると歯周病になりやすいことを知っていますか。

・□知っている　　　□知らない

8.     歯周病予防（歯科受診、健診、適切な歯磨き）をしたいと思いますか。

□とても思う　□すこし思う　□あまり思わない　□全く思わない

アンケートは以上になります。ご協力ありがとうございました。

Patient Questionnaire Survey Before the Study (English Version)

Questionnaire for "FollowNavi®" before this study

We are conducting a questionnaire to understand the current status before launching "FollowNavi®," a LINE-based service designed to provide information on periodontal disease prevention and conduct periodontal disease checks. Please take a moment to answer the following questions.

・The results of this survey will be presented at academic conferences and other events but will be published in a way that does not identify individuals.

・There will be no disadvantage to you if you do not respond to this questionnaire.

・You can withdraw your consent at any time.

Do you understand the contents of this survey and agree to participate?

□ I do not agree.

□ I agree. Please answer the following questions.　

Please check the box that best applies to each of the following questions.

1.    For how long have you been treated for diabetes?

□ Less than 1 year

□ 1 year to less than 5 years

□ 5 years to less than 10 years

□ 10 years or more

2.    Are you currently seeing a dentist?

□ Yes (Reason for visiting: __________________________________)

□ No

3.    Have you had a dental check-up (including dental caries and periodontal disease examinations, plaque removal, and brushing instruction) at a dental clinic within the past six months?

□ Yes (Frequency: _____ times)

□ No

4.    Have you ever consulted a pharmacist about your teeth or mouth?

□ Yes (Content: __________________________________)

□ No

5.    Do you try to keep your teeth healthy?

□ Yes (Specific actions: __________________________________)

□ No

6.    Do you know that having periodontal disease can worsen blood sugar levels?

□ Yes

□ No

7.    Do you know that diabetes is a risk for periodontal disease?

□ Yes

□ No

8.    Do you want to prevent periodontal disease (by consultation, the dentist, undergoing checkups, and brushing your teeth properly)?

□ Strongly agree

□ Agree

□ Disagree

□ Strongly disagree

Thank you for your cooperation.

Patient Questionnaire Survey After the Study (Japanese Version)

フォロナビ」使用後アンケート

フォロナビを使用した「LINEによる歯周病予防等に関する情報提供および歯周病のチェック」について、アンケート調査をしたいと思います。つきましては、下記のアンケートにご回答をお願いします。

※本調査の結果は、学会等で発表する予定ですが、個人が特定できない形で公表します。

※このアンケートにご回答いただかなくても不利益はありません。

※同意は当薬局に申し出ることにより、いつでも撤回することができます。

※この調査内容をご理解され、ご協力に同意いただけますか？

（調査にご協力いただける方は、「同意します」の方に必ず☑を入れてください）

□同意しません・・・この調査にはご協力いただかなくて結構です。

□同意します　・・・以下の設問にご回答ください。　

以下の質問に対して、当てはまるものに〇又は□に☑(チェック)を付けてください。

1.      現在、歯科受診をしていますか。

・□している（通院理由：　　　　　　　　　）　　□していない

2.      過去半年間に歯医者さんで検診（虫歯や歯周病の検査、歯垢除去、ブラッシングの指導）を受けたことがありますか。

・□受けた(頻度：年　　回)　　　□受けていない

3.      歯を健康に保つことを心掛けていますか。

・□ある　（具体的にやっていること：　　　　　　　　　）　□ない　

4.      歯周病があると血糖のコントロールが悪くなることを知っていますか。

・□知っている　　　□知らない

5.      糖尿病があると歯周病になりやすいことを知っていますか。

・□知っている　　　□知らない

6.      歯周病予防（歯科受診、検診、適切な歯磨き）をしたいと思いますか。

□とても思う　□すこし思う　□あまり思わない　□全く思わない

7.      過去半年間に薬剤師に、歯や口の中に関する相談をしたことがありますか。

□した　　□していない

8.      送られてくるLINEメッセージを見て、今後、歯の治療や検診に行こうと思いましたか。

□思った　　□少し思った　□あまり思わなかった　□全く思わなかった

9.      薬局から送られてくるLINEメッセージは、わかりやすかったですか？

１:わかりづらい　2:ややわかりづらい　3:どちらでもない　4:ややわかりやすい　5:わかりやすい

わかりづらい内容があった場合には、具体的に下記に記入してください。

（                                                                                                                                     ）

アンケートは裏面へ続きます

   10.   お薬のことでわからないことがあった場合に、LINEでのやり取りで疑問を解決することが出来ましたか？

1：LINEで解決することが出来た             2：LINE以外の方法で解決することが出来た

3：疑問を解決することが出来なかった      4：特にわからないことはなかった

LINEで解決出来なかった疑問があった場合には、具体的な内容を下記に記入してください。

（                                                                                                                              ）

11.   体調とお薬の使用状況の確認について、LINEからの質問の数はどうでしたか？

1：少ない　　2：やや少ない　　3：適切　　4：やや多い　　5：多い

12.   体調とお薬の使用状況の確認について、定期的に届くLINEからの質問の頻度はどうでしたか？

1：少ない　　2：やや少ない　　3：適切　　4：やや多い　　5：多い

13.   LINEによる体調とお薬の使用状況の確認において、1回あたりに、全ての質問に答えるのに要した時間はどれくらいでしたか？

1：5分以内　　　2：5～10分　　　3：10～20分　　　4：20～30分　　　5：30分以上

14.   今後も、このようなLINEによる体調確認を受けてみたいですか？

1：はい            2：いいえ

15.   LINEによる体調確認について、要望や改善点があればご記入ください。

（                                                                                                                              ）

アンケートは以上になります。ご協力ありがとうございました。

Patient Questionnaire Survey After the Study (English Version)

Questionnaire for "FollowNavi®" after this study

We are conducting a questionnaire to understand the current status after the study.

Please take a moment to answer the following questions.

・The results of this survey will be presented at academic conferences and other events but will be published in a way that does not identify individuals.

・There will be no disadvantage to you if you do not respond to this questionnaire.

・You can withdraw your consent at any time.

Do you understand the contents of this survey and agree to participate?

□ I do not agree.

□ I agree. Please answer the following questions.　

Please check the box that best applies to each of the following questions.

Are you currently seeing a dentist?

□ Yes (Reason for visiting: __________________________________)

□ No

Have you had a dental check-up (including dental caries and periodontal disease examinations, plaque removal, and brushing instruction) at a dental clinic within the past six months?

□ Yes (Frequency: _____ times/year)

□ No

Do you try to keep your teeth healthy?

□ Yes (Specific actions: __________________________________)

□ No

4.    Do you know that diabetes is a risk for periodontal disease?

□ Yes

□ No

Do you know that people with diabetes are more likely to develop periodontal disease?

□ Yes

□ No

Do you want to prevent periodontal disease (by consultation, the dentist, undergoing checkups, and brushing your teeth properly)?

□ Strongly agree

□ Agree

□ Disagree

□ Strongly disagree

Have you ever consulted a pharmacist about your teeth or mouth?

□ Yes

□ No

Did you consider contacting a dentist by message?

□ Strongly agree

□ Agree

□ Disagree

□ Strongly disagree

Did you understand the text messages well?

□ Well understood

□ Understood

□ Neutral

□ Slightly difficult

□ Difficult

If you found any part difficult to understand, please explain: (_______________)

Did you solve your question with "FollowNavi®"?

□ Solved with ‟FollowNavi®”

□ Solved in another way

□ No

□ No question

 If you have any questions that could not be resolved via LINE, please write the specific details below. (______________________________________________________)

Was the number of questions appropriate?

□ Small

□ Slightly small

□ Appropriate

□ Slightly large

□ Large

Was the frequency of questions appropriate?

□ Low

□ Slightly low

□ Appropriate

□ Slightly high

□ High

How long did it take to answer the question?

□ Within 5 min

□ 5-10 min

□ 10-20 min

□ 20-30 min

□ Over 30 min

Do you want to continue using "FollowNavi®"

□ Yes

□ No

Do you have any requests or suggestions for improving the LINE health check? (______________________________________________________)

Thank you for your time and cooperation.

Pharmacist Questionnaire Survey (Japanese Version)

【薬剤師向け】「フォロナビ」によるフォローアップ実施に関するアンケート

「フォロナビ」による服薬期間中のフォローアップの取り組みについて、薬剤師の方にアンケート調査したいと思います。つきましては、下記のアンケートにご回答いただきたく、ご協力をお願いいたします。

※本調査の結果は学会等で発表する予定ですが、個人が特定できない形で公表します。

※このアンケートにご回答いただかなくても不利益を被りません。

※同意は当薬局に申し出ることにより、いつでも撤回することができます。

※この調査内容をご理解され、ご協力に同意いただけますか？

（調査にご協力いただける方は、「同意します」の方に必ず☑を入れてください）

□同意しません・・・この調査にはご協力いただかなくて結構です。

□同意します　・・・以下の設問にご回答ください（あてはまるものに〇をしてください）。　

1.       今回の取り組みで、「フォロナビ」を用いて服薬期間中のフォローアップを実施しましたか？

１：はい（以下の設問にもお答えください）　　　　　２：いいえ

2.       今回の取り組み以前に、糖尿病患者から歯科に関する相談を受けたことがありますか？

１：ある （相談内容：　　　　　　　　　　　　　　　　　　　　　）　　　　　２：ない

3.       今回の取り組み中に、研究対象患者から来局時に歯や口の中に関する相談を受けましたか？

１：受けた （相談内容：　　　　　　　　　　　　　　　　　　　　）　　　　　２：受けていない

4.       「フォロナビ」から患者に自動的に送信するメッセージは患者にとって分かりやすい内容だと思いますか？

１：分かりづらい　2：やや分かりづらい　3：どちらでもない　4：やや分かりやすい　5：分かりやすい

分かりづらいと思う表現がありましたら具体的にご記入ください

（                                                                                                                                     ）

5.       「フォロナビ」によるやり取りで患者の情報を十分に得ることが出来ましたか？

1：はい                2：いいえ　

フォロナビによるやり取りで得ることが出来なかった情報があった場合は具体的にご記入ください。

（                                                                                                                              ）

6.       「フォロナビ」によるフォローアップについて、患者への質問の数はどうでしたか？

1：少ない　　2：やや少ない　　3：適切　　4：やや多い　　5：多い

7.       「フォロナビ」によるフォローアップについて、フォローアップの頻度はどうでしたか？

1：少ない　　2：やや少ない　　3：適切　　4：やや多い　　5：多い

8.       「フォロナビ」の操作は使いやすかったですか？

1：使いづらい　　2：やや使いづらい　　3：どちらでもない　　4：やや使いやすい　　5：使いやすい

使いづらい点がありましたら具体的にご記入ください。

アンケートは裏面へ続きます

（                                                                                                                              ）

9.       「フォロナビ」によるフォローアップの内容確認1回に要した時間はどれくらいでしたか？

1：5分以内　　　　2：5～10分　　　　3：10～20分　　　　4：20～30分　　　　5：30分以上

10.    今後、「フォロナビ」を使用することで期待できる効果を選択して下さい(複数回答可)。

□患者の症状確認　　　□アドヒアランス向上　　　□副作用早期発見　　　□治療効果向上　

□指導時間短縮(電話指導と比較して)　　　□その他（                                                       ）

11.    今後も、「フォロナビ」による服薬期間中のフォローアップを行っていきたいですか？

1：はい                2：いいえ

12.    「フォロナビ」による服薬期間中のフォローアップについて、要望や改善点があればご記入ください。

（                                                                                                                              ）

アンケートは以上になります。ご協力ありがとうございました

Pharmacist Questionnaire Survey (English Version)

Questionnaire for "FollowNavi®" for pharmacists

We would like to conduct a questionnaire for pharmacists regarding the follow-up activities during the medication period using "FollowNavi®." Please take a moment to answer the following questions.

・The results of this survey will be presented at academic conferences and other events but will be published in a way that does not identify individuals.

・There will be no disadvantage to you if you do not respond to this questionnaire.

・You can withdraw your consent at any time.

Do you understand the contents of this survey and agree to participate?

□ I do not agree.

□ I agree. Please answer the following questions.　　

Please check the box that best applies to each of the following questions.

Did you use "FollowNavi®"?

□ Yes

□ No

Have you been asked questions about dentistry before this trial?

□ Yes

□ No

Did you have any questions about dentistry during this trial?

□ Yes (Consultation content: _________________________________________)

□ No

Do you think the text messages are easy for patients to understand?

□ Strongly agree

□ Agree

□ Disagree

□ Strongly disagree

If you thought any expressions were difficult to understand, please specify:

(____________________________________________________________________)

Did you get sufficient patient information?

□ Yes

□ No

If there was any information that you were unable to obtain through interactions with "FollowNavi®", please specify: (____________________________________________)

Was the number of questions appropriate?

□ Small

□ Slightly small

□ Appropriate

□ Slightly large

□ Large

Was the frequency of questions appropriate?

□ Low

□ Slightly low

□ Appropriate

□ Slightly high

□ High

How easy was "FollowNavi®" to use?

□ Easy

□ Slightly easy

□ Neutral

□ Slightly difficult

□ Difficult

If there were any difficulties you encountered, please specify: (______________________)

How long did it take to answer the question?

□ Within 5 min

□ 5-10 min

□ 10-20 min

□ 20-30 min

□ Over 30 min

Please select the expected effects of using "FollowNavi®" in the future (multiple answers possible).

□ Confirmation of patient symptoms

□ Improved adherence

□ Early detection of adverse drug reactions

□ Improving therapeutic effect

□ Reduction of medication instruction time

□ Other (___________________________________________)

Do you want to continue using "FollowNavi®"?

□ Yes

□ No

If you have any requests or suggestions for improvement regarding follow-up using "FollowNavi®", please describe them below. (_____________________________________)

Thank you for your cooperation.
